# Down-regulation of SOSTDC1 promotes thyroid cancer cell proliferation via regulating cyclin A2 and cyclin E2

**DOI:** 10.18632/oncotarget.5566

**Published:** 2015-09-10

**Authors:** Weiwei Liang, Hongyu Guan, Xiaoying He, Weijian Ke, Lijuan Xu, Liehua Liu, Haipeng Xiao, Yanbing Li

**Affiliations:** ^1^ Department of Endocrinology and Diabetes Center, The First Affiliated Hospital of Sun Yat-sen University, Guangzhou, Guangdong, China

**Keywords:** SOSTDC1, thyroid cancer, proliferation, cyclin A2, cyclin E2

## Abstract

Sclerostin domain containing protein 1 (SOSTDC1) is down-regulated and acts as a tumor suppressor in some kinds of cancers. However, the expression pattern and biological significance of SOSTDC1 in thyroid cancer are largely unknown. We demonstrated that SOSTDC1 was significantly down-regulated in **thyroid** cancer. Ectopic over-expression of SOSTDC1 inhibited proliferation and induced G1/S arrest in thyroid cancer cells. Moreover, SOSTDC1 over-expression suppressed the growth of tumor xenografts in nude mice. We also found that elevated SOSTDC1 led to inhibition of cyclin A2 and cyclin E2. Together, our results demonstrate that SOSTDC1 is down-regulated in thyroid cancer and might be a potential therapeutic target in the treatment of thyroid cancer.

## INTRODUCTION

Thyroid cancer is currently the most common endocrine malignancy, and its incidence is rising fast [1]. Thyroid neoplasia is histologically classified into differentiated papillary carcinoma (PTC), follicular carcinomas (FTC) and undifferentiated anaplastic carcinoma (ATC). Molecular genetic studies have demonstrated that thyroid carcinogenesis involves gradual accumulation of a series of genetic and epigenetic alterations, leading to gain-of-function mutations in oncogenes and loss-of-function mutations in tumor suppressor genes [2, 3]. Consequently, there is a need for exploration of the genetic events involved in thyroid cancer initiation and progression to improve our understanding of thyroid tumorigenesis and develop effective therapeutic strategies.

SOSTDC1 (sclerostin domain containing protein 1, also known as WISE, USAG1, ectodin) functions as antagonist of bone morphogenetic proteins (BMP) and Wnt signaling [4]. SOSTDC1 participates in multiple developmental processes including the teeth [5], hair [6], limb [7] and head [8]. SOSTDC1 also plays a vital role in the development of various cancers, such as breast cancer [9], renal tumors [10], Wilms tumor [11] and gastric cancer [12]. For example, it has been reported that SOSTDC1 is significantly down-regulated in clear cell renal carcinoma and over-expression of SOSTDC1 inhibits proliferation of clear cell carcinoma cells through regulating both BMP and Wnt signaling [10]. Recently, Gopisetty *et al*. found that SOSTDC1 was down-regulated in gastric tumors, and acted as a tumor suppressor in gastric cancer [13]. However, the expression and biologic function of SOSTDC1 in thyroid cancer remains unclear.

The retinoblastoma tumor suppressor protein (pRb), which has been identified as a human tumor suppressor, plays an integral role in the regulation of cancer cell cycle progression [14-16]. pRb controls the G1/S boundary, in large part by repressing the transcriptional activity of the E2F transcription factors, which binds with pRb. Many growth factors lead to a significant phosphorylation of pRb, resulting in its inactivation. Inactivated pRb can release and active E2Fs. Free E2Fs function as transcription factors can induce the expression of many genes involved in G1/S transition [17]. However, little is known regarding the function and regulation mechanisms of Rb-E2F pathway in thyroid cancer.

In this study, we show that SOSTDC1 is down-regulated in thyroid cancer. Moreover, our data suggest that SOSTDC1 inhibits the proliferation of thyroid cancer cells via regulation of cyclin A2 and cyclin E2.

## RESULTS

### SOSTDC1 is clinically associated with the Rb-E2F pathway

**Figure 1:**
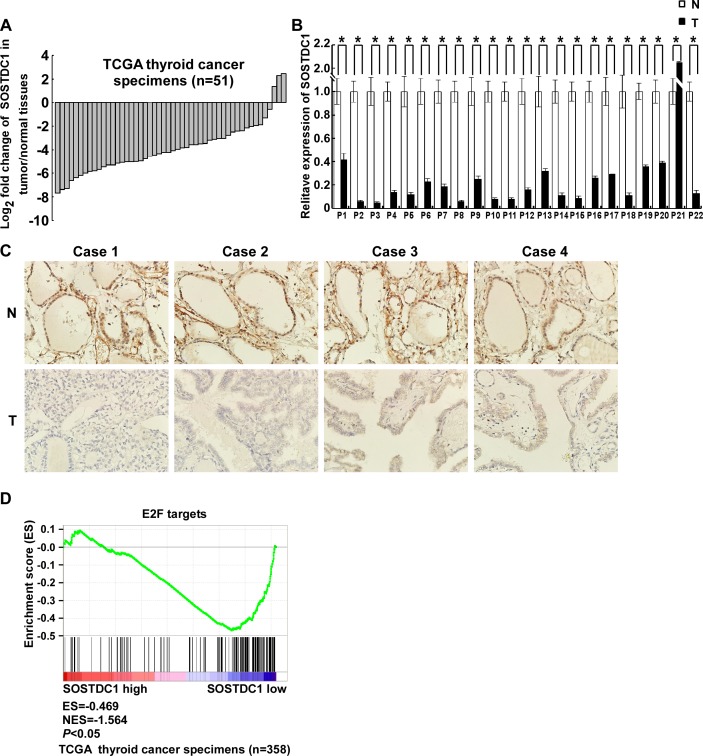
SOSTDC1 is clinically associated with the Rb-E2F pathway **A.** The expression of SOSTDC1 in 51 pairs of primary tumors versus paired non-tumorous thyroid tissues using RNAseqV2 data sets deposited on TCGA website. **B.** Expression of SOSTDC1 in 22 paired tumors and adjacent non-tumorous thyroid tissues assessed by qRT-PCR. **C.** Expression of SOSTDC1 in 12 paired tumors and adjacent non-tumorous thyroid tissues assessed by IHC. **D.** GSEA plotting shows that SOSTDC1 expression is negatively correlated with a signature of genes responsive to E2F activation (TCGA dataset, *n* = 358).

We analyzed the expression of SOSTDC1 in 51 pairs of thyroid tumors and their adjacent non-tumorous thyroid tissues using RNAseqV2 data sets for thyroid cancer deposited on The Cancer Genome Atlas (TCGA, https://tcga-data.nci.nih.gov/tcga/) website. The results showed that SOSTDC1 expression was attenuated in thyroid cancer tissues compared to adjacent non-tumorous thyroid tissues (Figure 1A). To further validate the expression of SOSTDC1 in thyroid cancer, we used real-time polymerase chain reaction (PCR) to determine the expression levels of SOSTDC1 in 22 pairs of tumors and their adjacent non-tumorous thyroid tissues. In agreement with the TCGA data, the expression levels of SOSTDC1 were significantly decreased in most thyroid tumor tissues in comparison with those in adjacent non-tumorous thyroid tissues (Figure 1B). To study the protein expression of SOSTDC1, immunohistochemistry (IHC) was performed in 12 pairs of tumors and their adjacent non-tumorous thyroid tissues. The results showed that SOSTDC1 expression was significantly down-regulated in tumors as compared with their adjacent non-tumorous tissues (Figure 1C). These results imply that down-regulation of SOSTDC1 may play a biologic role in human thyroid cancer disease processes.

**Figure 2:**
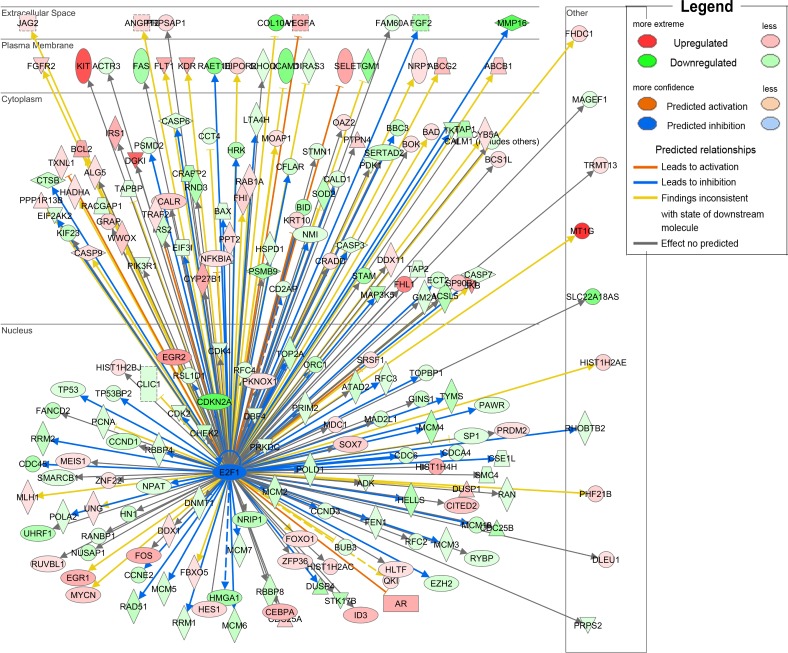
URA analysis reveals that the transcriptional factor E2F was the predicted upstream regulator responsible for the differential expression of genes identified in low and high SOSTDC1 expression samples

To further investigate the functional influences and the involved pathways of down-regulated SOSTDC1 on thyroid cancer, we downloaded RNAseqV2 data sets for 358 cases of thyroid cancers from the TCGA data portal. We analyzed the fold differences in transcript abundance between low and high SOSTDC1 expression (median as cutoff) used MultiExperiment Viewer (MeV, http://www.tm4.org/mev.html) software. Then the significantly differentially expressed genes were imported into the Ingenuity Pathway Analysis (IPA) software (Ingenuity Systems, http://www.ingenuity.com/) and analyzed. The analysis indicated that “cellular growth and proliferation” was the top biological function mediated by these differentially expressed genes identified in low and high SOSTDC1 expression samples. Furthermore, to find out the altered upstream regulators that could be responsible for the observed expression changes, we performed IPA Upstream Regulator Analysis (URA). URA could predict whether the upstream regulators are activated or inhibited based on the distinct up- and down-regulation pattern of the expressed genes, and determine which causal relationships previously reported in the literature are likely relevant for the biological mechanisms underlying the data. A Z-score>2 was considered to be activated, and Z-score <-2 was considered to be inhibited. URA analysis revealed that the transcriptional factor E2F, with a negative Z-score, was one of the top predicted upstream regulators. As it is well known that E2F widely participates in the development of various cancers, we focused on studying whether E2F is involved in the effect of SOSTDC1 on the thyroid cancer cells (Figure 2).

We further carried out gene set enrichment analyses (GSEA, http://www.broadinstitute.org/gsea/index.jsp) to investigate whether E2F up-regulated genes are enriched among the thyroid cancer patients with low SOSTDC1 expression. As shown in Figure 1D, E2F signature was significantly enriched in low SOSTDC1 expression group, in comparison with high expression group, indicating preferential E2F activation in thyroid cancer specimens with low SOSTDC1 expression. Taken together, these results indicate that SOSTDC1 may inhibit thyroid tumorigenesis, at least partly, through regulating the Rb-E2F pathway.

### SOSTDC1 is down-regulated in human thyroid cancer tissue

**Figure 3:**
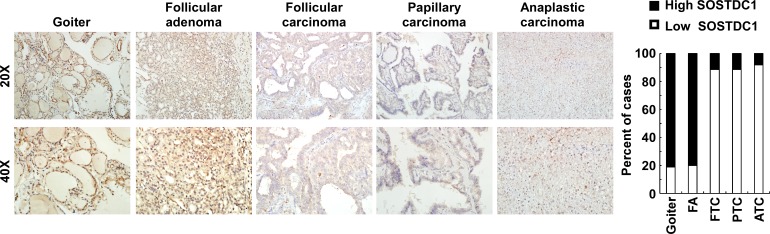
SOSTDC1 is down-regulated in human thyroid cancer tissue **A.** Representative images of IHC assays on SOSTDC1 expression in thyroid lesions. **B.** Percentage of specimens showing low or high SOSTDC1 expression in thyroid lesions.

To evaluate the clinical relevance of SOSTDC1 in thyroid cancer, we investigated the SOSTDC1 protein expression in 16 cases of goiters, 15 cases of adenomas, and 107 cases of thyroid cancers (26 cases FTC, 69 cases PTC, and 12 cases ATC) by immunohistochemistry. As shown in Figure 3, the expression level of SOSTDC1 protein in thyroid malignant lesions was significantly decreased as compared with that in benign lesions (goiters and adenomas). Interestingly, low expression of SOSTDC1 was detected in 88.8% thyroid cancer (95/107), whereas SOSTDC1 is highly expressed in most goiter (13/16) and adenoma specimens (12/15). Of note, no significant difference was observed in the protein levels of SOSTDC1 in FTC, PTC, and ATC, implying that down-regulation of SOSTDC1 in thyroid cancers did not correlate with the grade of malignancy. To further evaluate the role of SOSTDC1 in thyroid cancer progression, we analyzed the correlation between SOSTDC1 expression and clinical features of patients. As summarized in Table 1, there was significant correlation between SOSTDC1 expression and tumor-node-metastasis staging (p =0.008). In addition, SOSTDC1 expression was significantly correlated with the tumor size (p=0.038). The SOSTDC1 expression levels did not differ significantly by age (p =0.161) and gender (p =0.299). Taken together, these data suggested that SOSTDC1 is down-regulated in thyroid cancer and the altered expression of SOSTDC1 may play an important role in the process of cancer progression.

**Table 1:** Association between clinicopathologic parameters and level of SOSTDC1 protein expression in thyroid cancer patients

Clinicopathologic variables	SOSTDC1		*P* value
	Low	High	
Age			
<45 yr	51	9	0.161
≥45 yr	44	3	
Gender			
Male	22	5	0.299
Female	73	7	
TNM stage			
I and II	49	11	0.008
III and IV	46	1	
T classification			
T1 and T2	53	11	0.038
T3 and T4	42	1	
N classification			
N0	46	8	0.083
N1	49	4	

SOSTDC1, sclerostin domain containing protein 1.

### Ectopic over-expression of SOSTDC1 inhibits proliferation and induces G1/S arrest in thyroid cancer cells

**Figure 4:**
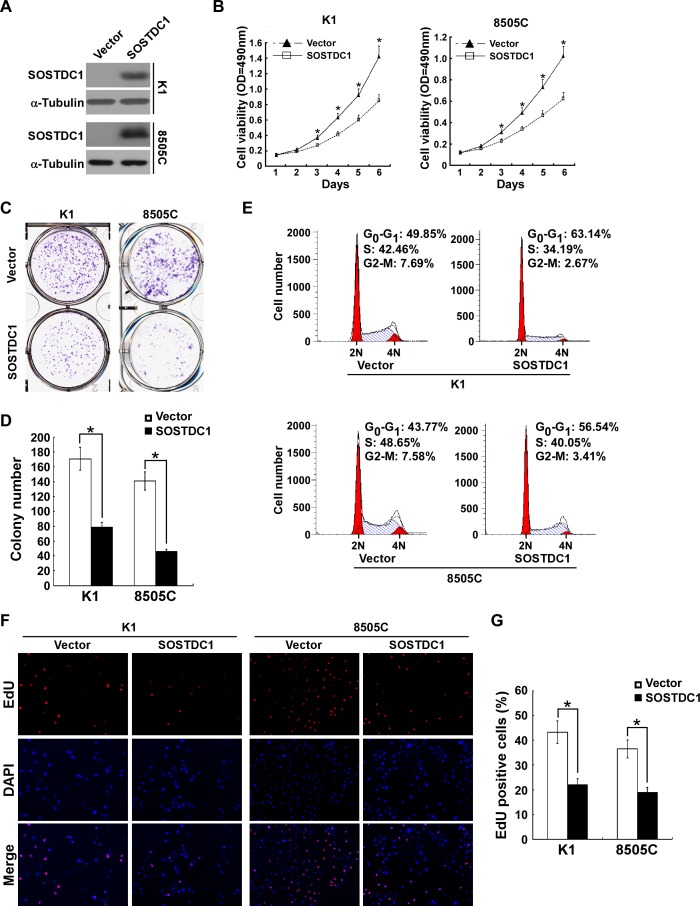
Ectopic over-expression of SOSTDC1 inhibits the proliferation of thyroid cancer cells **A.**Protein expression of SOSTDC1 in indicated cells was analyzed by Western blotting assay. α-tubulin was used as a loading control. **B.** MTT assay was conducted to investigate the effect of SOSTDC1 on the proliferation of indicated thyroid cancer cells at the indicated time points. **C.** and **D.** Representative micrographs **C.** and relative quantification **D.** of colony formation assays of indicated cells. **E.** Flow-cytometric determination of proportion of indicated the studied cells in distinct cell-cycle phases. **F.** and **G.** Representative images **F.** and relative quantification **G.** of EdU incorporation assays. For **B.**, **D.**, and **G.**, the data are reported as mean ± SD of 3 independent experiments. *P < 0.05.

To investigate the functional role of SOSTDC1 in thyroid cancer progression, we established two stably expressed SOSTDC1 thyroid cancer cell lines, K1/SOSTDC1 and 8505C/SOSTDC1 (Figure 4A). Next, we evaluated the cell viability by MTT assay. As shown in Figure 4B, ectopic expression of SOSTDC1 decreased the growth of both K1 and 8505 cells as compared with vector-control cells. Our data from colony formation assay demonstrate that SOSTDC1 over-expressing cells exhibited fewer colonies compared with control cells (Figure 4C and 4D). As shown by flow cytometry analysis in Figure 4E, the SOSTDC1 over-expression resulted in an elevated in G1-phase proportion and a reduced in G2/M phase proportion. These results suggest that SOSTDC1 plays an important role in cell cycle control for thyroid cancer cells. Moreover, we performed EdU assay to assess the influence of SOSTDC1 to the DNA synthesis. The results showed that the percentage of EdU-positive cells was lower on average in cells which were transfected with SOSTDC1 (Figure 4F and 4G). Collectively, these data indicate that SOSTDC1 inhibits proliferation of thyroid cancer cells.

### SOSTDC1 inhibits the expression of cyclin A2 and cyclin E2

**Figure 5:**
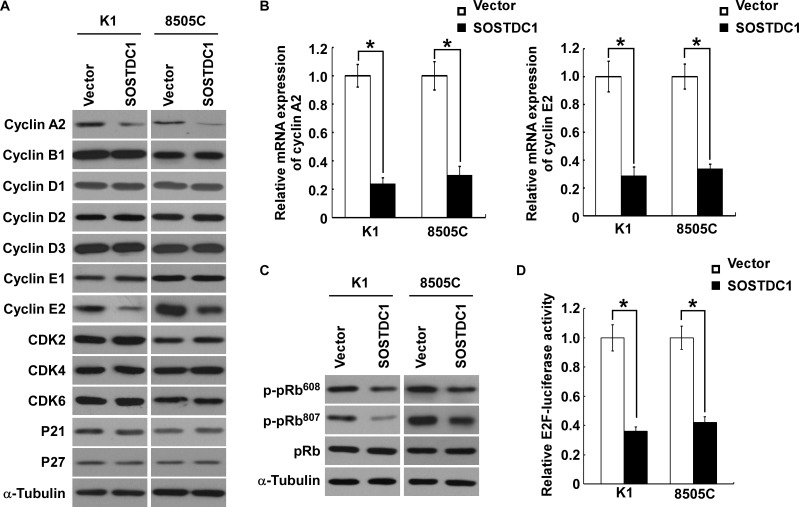
Ectopic over-expression of SOSTDC1 inhibits the expression of cyclinA2 and cyclin E2, and suppresses E2F transcriptional activity **A.** Western blotting analysis was performed to detect the cell cycle regulator cyclin A2, cyclin B1, cyclin D1, cyclin D2, cyclin D3, cyclin E1, cyclin E2, CDK2, CDK4, CDK6, p21^Cip1^, and p27^Kip1^ in indicated cells. α-tubulin was used as a loading control.**B.** qRT-PCR was conducted to detect the mRNA expression levels of cyclin A2 and cyclin E2 in indicated cells. GAPDH was used as a house keeping gene control. **C.** Ectopic expression of SOSTDC1 in the studied cells significantly inhibited the phosphorylation of pRb at Ser608 and Ser807 residues. α-tubulin served as the sample loading control. **D.** Over-expression of SOSTDC1 attenuates E2F transcriptional activity using E2F-luc reporter assay. For **B.** and **D.**, results derived from three independent experiments are expressed as mean ± SD. *P < 0.05.

To understand specific mechanisms underlying the inhibitory effect of SOSTDC1 on thyroid cancer cell proliferation, we examined the protein expression level of cell cycle regulators by Western blot analysis. As illustrated in Figure 5A, SOSTDC1 over-expression decreased the levels of cyclin A2 and cyclin E2 proteins in K1 and 8505C cells, but no changes in protein levels of cyclin B1, cyclin D1, cyclin D2, cyclin D3, cyclin E1, CDK2, CDK4, CDK6, p21^Cip1^, and p27^Kip1^ were observed. Moreover, over-expression of SOSTDC1 led to reduction of mRNA expression levels of cyclin A2 and cyclin E2 (Figure 5B). It is well known that Rb-E2F pathway is the main substrates of CDK/cyclin complexes, and the URA results show that *SOSTDC1* is clinically associated with the Rb-E2F pathway. So we further examined the effects of SOSTDC1 on pRb phosphorylation and E2F transcriptional activity. As expected, over-expression of SOSTDC1 decreased the phosphorylation of pRb at Ser608 and Ser807 residues (Figure 5C) and E2F transcriptional activity (Figure 5D). Taken together, these results indicate that SOSTDC1 suppresses proliferation through inhibiting the expression of cyclin A2 and cyclin E2 proteins, and the activity of Rb-E2F pathway.

### SOSTDC1 suppresses tumor growth *in vivo*

**Figure 6:**
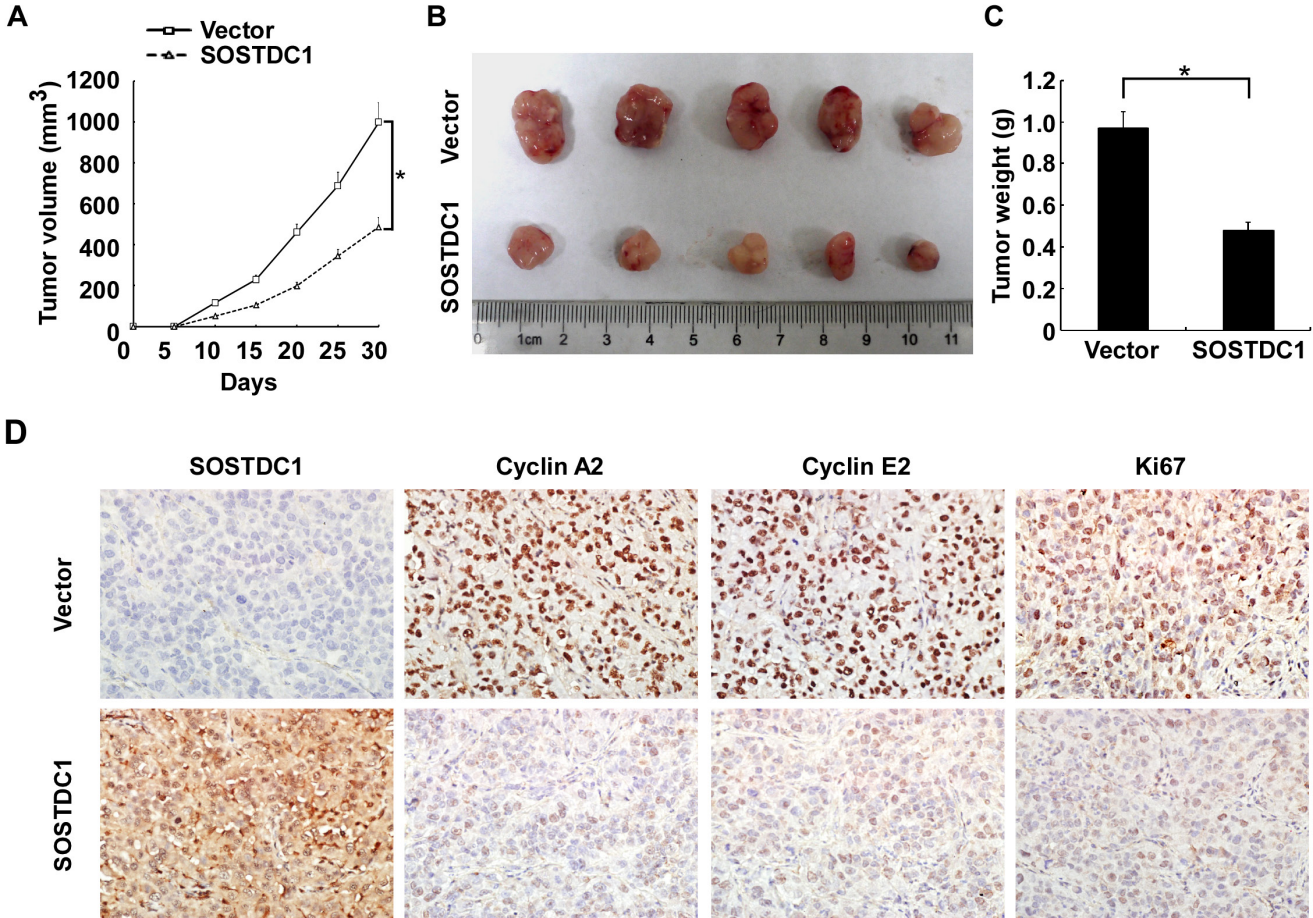
SOSTDC1 suppresses tumor growth *in vivo* **A.** Quantitative analysis of tumor volumes. **B.** Representative image of subcutaneous tumors isolated from nude mice. **C.** Quantitative analysis of tumor weights. The indicated tumor volumes and weights represent the mean ± SD of five animals in each group. **D.** Representative images of IHC assays on SOSTDC1, cyclin A2, cyclin E2 and Ki67 expression in resected tumor specimens.* p<0.05.

To investigate the biological effect of ectopic over-expression of SOSTDC1 on tumor growth in vivo, we proceeded with the research through the establishment of a subcutaneous xenograft tumor model in nude mice. The growth curve revealed a dramatic decrease of tumor size in the group with SOSTDC1 over-expression (Figure 6A). As shown in Figure 6B and 6C, the tumor size and weight of the SOSTDC1 over-expression group was smaller than that of control group. IHC staining showed that ectopic over-expression of SOSTDC1 significantly inhibited the expression of Ki67, a well-established cell proliferation marker. In addition, consistent with the results of cell proliferation assays, cyclin A2 and cyclin E2 were also down-regulated following SOSTDC1 over-expression (Figure 6D). Therefore, SOSTDC1 inhibits the growth of thyroid cancer cells in vivo, likely by decreasing cyclin A2 and cyclin E2.

## DISCUSSION

The current study demonstrated that SOSTDC1 expression was significantly reduced in thyroid cancer. Moreover, ectopic over-expression of SOSTDC1 led to down-regulation of cyclin A2 and cyclin E2, which subsequently inhibit thyroid cancer cell proliferation in vitro and in vivo. Our results underline a fundamental role of SOSTDC1 as a tumor suppressor in thyroid cancer.

An increasing number of studies have demonstrated that SOSTDC1 is dysregulated and acts as a tumor suppressor in some kinds of human cancers. For example, it was previously indicated that SOSTDC1 is significantly down-regulated in clear cell renal carcinoma and over-expression of SOSTDC1 inhibits its proliferation through suppressing BMP-7-induced phosphorylation of Smad-1, -5, and -8, as well as Wnt-3a signaling [10]. Moreover, Blish KR *et al.* confirmed that loss of heterozygosity for SOSTDC1 has been identified in adult and pediatric renal tumors [18]. In Wilms tumor, SOSTDC1 has been identified as a candidate tumor suppressor gene, which may have a role in the progression of tumorigenesis [11]. Recently, it was demonstrated that SOSTDC1 protein expression was down-regulated in gastric tumors, and ectopic over-expression in gastric cancer cells inhibits their tumorigenic properties [13]. Indeed, Barderas *et al.* reported that SOSTDC1 was up-regulated in highly metastatic KM12SM colorectal cancer cells, and silencing of SOSTDC1 caused a significant decrease in migration, invasion, and metastasis [19]. In our present study, for the first time SOSTDC1 was found to be down-regulated and functioned as a tumor suppressor gene through inhibiting cell proliferation in thyroid cancer. However, the mechanisms involved in down-regulation of SOSTDC1 in thyroid cancer are largely unknown. Recently, down-regulation of SOSTDC1 expression by epigenetic mechanisms, such as methylation, has been documented in cancer. Gopisetty *et al.* observed that SOSTDC1 methylation can inhibit SOSTDC1 promoter activity and suppress gene expression in gastric cancer [13]. In breast cancer, methylation also participated in the down-regulation of SOSTDC1. Akhilesh *et al.* showed that the transcriptional repressor E4BP4 epigenetically repressed SOSTDC1 expression in breast cancer cells [20]. Thus, it is likely that epigenetic mechanisms might also play a role in down-regulation of SOSTDC1 in thyroid cancer.

We also further explored the potential mechanism by which SOSTDC1 suppresses thyroid cancer cell proliferation. The results showed that over-expression of SOSTDC1 in thyroid cancer cells resulted in dys-regulating the expression of critical cell cycle regulators, cyclin A2 and cyclin E2 in vitro and vivo. Moreover, consistent with the results of biologic analysis, we found that Rb-E2F pathway also involved in the anti-proliferative role.

Cyclins are activators of specific serine/threonine protein kinases, which can promote the cell cycle process. Cyclin A2, which is particular in the cyclin family, activates two different cyclin-dependent kinases (CDKs) and promotes both G1/S and G2/M phase transitions [21]. Consistent with its role as a key cell cycle regulator, over-expression of cyclin A2 is found in many cancers, including anaplastic thyroid carcinoma [22]. In addition, over-expression of cyclin A2 in cancers is associated with enhanced cellular proliferation and predicted a poor prognosis [23]. Cyclin E2, which can bind to Cdc2-related kinase (CDK2), plays a critical role for G1/S transition. It aberrantly expresses in human cancers [24], and its over-expression is associated with proliferation in various cancers. For example, the levels of cyclin E2 was significantly elevated in human breast cancers [25], and decrease of cyclin E2 severely attenuates the estrogen-induced breast cancer cell proliferation [26]. Moreover, the high expression level of Cyclin E2 was related to a poor prognosis in ER-positive breast cancer [27, 28]. Cyclin E2 is also over-expressed in several small lung cancer cell lines [24], and ectopic over-expression of cyclin E2 reversed the G0/G1 cell cycle arrest induced by miR-25 down-regulation [29]. Similar with the previous studies, our data demonstrated that decreased cyclin A2 and cyclin E2 were involved in the anti-proliferative role of SOSTDC1 in thyroid cancer.

To further address the molecular mechanisms involved in SOSTDC1-mediated inhibition of proliferation, Rb-E2F pathway was selected for study. Because it is well known that Rb-E2F pathway is the main substrate of CDK/cyclin complexes, and the URA results show that SOSTDC1 is clinically associated with the Rb-E2F pathway. Emerging evidence has shown that Rb is a tumor suppressor and plays a critical role in regulating G1/S transition and cell proliferation. Rb exerts its growth regulatory properties, in large part by repressing the transcriptional activity of the E2F transcription factors. Rb-E2F pathway plays an important role in regulating tumor cell proliferation [30]. However, little is known about the function of Rb-E2F pathway in thyroid cancer. Our study demonstrated that over-expression of SOSTDC1 significantly inhibited the phosphorylation of pRb and E2F transcriptional activity.

In conclusion, our data demonstrate that SOSTDC1 is down-regulated in thyroid tumor tissues and inhibits thyroid cancer cell proliferation through modulating cyclin A2 and cyclin E2. Given these findings, SOSTDC1 might be a potential therapeutic target in the treatment of thyroid cancer.

## MATERIALS AND METHODS

### Cell culture

Human thyroid cancer cell lines, K1 and 8505C cells were obtained from the European Collection of Cell Cultures (ECACC, Salisbury, United Kingdom) and maintained in DMEM with 10% FBS.

### Vectors and retroviral infection

A SOSTDC1 construct was generated by subcloning polymerase chain reaction (PCR)-amplified full-length human SOSTDC1 cDNA into pQCXIP (Clontech, Palo Alto, CA). For retroviral transduction, Pt67 (Clontech, Palo Alto, CA) was incubated with a precipitated mixture of Lipofectamine 2000 (Life Technologies, Gaithersburg, MD) and pQCXIP-SOSTDC1 plasmid for 24 h [31]. Stable cell lines were selected by treatment with 0.5μg/ml puromycin for 10 days, beginning 48 hours after infection.

### Patients and tissue specimens

A total of 138 cases of paraffin-embedded thyroid tissue (26 cases of FTC, 69 cases of PTC, and 12 cases of ATC, 15 cases of adenoma samples, and 16 cases of goiters) and 22 pairs of human thyroid cancer tissues and their adjacent non-cancerous thyroid tissues were obtained from the First Affiliated Hospital of Sun Yat-sen University between 2008 to 2013, with the approval of the Institutional Research Ethics Committee. The pathology of all tissue parts was confirmed by two pathologists. The 22 pairs of tissue specimens were collected, frozen, and stored in liquid nitrogen until used.

### MTT assay

Cell growth was determined using a MTT assay. The cells were seeded at a density of 5×10^3^ cells per well in 96-well plates. Then at 1, 2, 3, 4, 5 and 6 days, 20 μl MTT (Sigma-Aldrich, St. Louis, MO) was added to each well and incubated for 4 h. The culture medium was removed and 200 μl dimethyl sulfoxide (DMSO) (Amresco, Solon, Ohio) was added to each well. The plates were then shaken for 30 minutes, and the optical density (OD) at 490 nm was measured using an ELISA reader. Each experiment was performed in triplicate.

### Colony formation assay

For the colony formation assay, cells were plated at 500 per well into 6-well plates. The cells were allowed to grow 10 days and stained with crystal violet. The plates were photographed and the numbers of colonies formed by indicated cells were quantified using the Quantity One software package (Bio-Rad, Hercules, CA). Each experiment was repeated three times.

### Cell cycle analysis

For cell cycle analysis, cells were harvested by trypsinization and washed twice with ice-cold phosphate-buffered saline (PBS). The cells were then fixed in 75% ethanol, treated with RNase A (Sigma-Aldrich, St. Louis, MO), followed by incubation with propidium iodide (Sigma-Aldrich, St. Louis, MO). Cell cycle analysis was determined using a flow cytometer (Beckman-Coulter, Hialeah, FL).

### 5-ethynyl-2′-deoxyuridine (Edu) incorporation assay

To determine the DNA synthesis, the Cell Light EdU DNA imaging kit (RiboBio Co., Guangzhou, China) was used. In short, cells were seeded in 24-well plates and exposed to EdU for 2 h. Then they were fixed in 4% paraformaldehyde and permeabilized in 0.5% Triton X-100. Images were taken using a fluorescent microscope at 488 nm excitation. Each experiment was repeated independently thrice.

### Western blotting

Western blotting was performed according to a standard method as described previously [32]. Antibodies for immunoblotting were as follows: anti-SOSTDC1, anti-cyclin B1, anti-cyclin D3 (Abcam, Cambridge, MA), anti-cyclin A2, anti-cyclin D1, anti-cyclin E1, anti-CDK4, anti-CDK6 (Epitomics, Burlingame, California), anti-cyclin D2, anti-CDK2 (BD Pharmingen, San Diego, CA), anti-cyclin E2, anti-p21^Cip1^, anti-p27^Kip1^, anti-p-pRb Ser608, anti-p-pRb Ser807, anti-pRb (Cell Signaling, Beverly, MA) and anti-α-tubulin (Sigma-Aldrich, St. Louis, Missouri).

### RNA extraction and real-time reverse transcription-polymerase chain reaction

Total RNA from tissues was extracted by TRizol reagent (Life Technologies, Gaithersburg, MD), and reverse transcription (RT) reactions and real-time polymerase chain reaction (PCR) were performed as described previously [33]. The following primers were used: SOSTDC1 forward, 5′-CACGTTGAATCAAGCCAGAA-3′ and reverse, 5′-GATGTATTTGGTGGAACGCA-3′; cyclin A2 forward, 5′-CAGAAAACCATTGGTCCCTC-3′ and reverse, 5′-CACTCACTGGCTTTTCATCTTC-3′; cyclin E2 forward, 5′-ACCTCATTATTCATTGCTTCCAA-3′ and reverse, 5′-TCTTCACTGCAAGCACCATC-3′; and GAPDH forward, 5′-GACTCATGACCACAGTCCATGC-3′ and reverse, 5′-AGAGGCAGGGATGATGTTCTG-3′.

### Luciferase reporter assay

Dual-Luciferase reporter assays (Promega, Madison, Wisconsin) were performed according to the manufacturer's instructions as previously described [33]. Briefly, K1 and 8505C cells were seeded in triplicate in 24-well plates and allowed to settle for 24 h. Co-transfection of pE2F-TA-Luc plasmid (Clontech, San Francisco, CA) and 1 ng pRL-TK Renilla was performed using Lipofectamine 2000 Reagent (Life Technologies, Gaithersburg, MD) according to the manufacturer's protocol. Thirty six hours after transfection, the cells were harvested and lysed, and the luciferase activities were assessed. The firefly luciferase activities were normalized to Renilla luciferase activities. Three independent experiments were performed.

### *In vivo* experiments

Five female BALB/c mice (4 weeks old) were used to assess the effect of SOSTDC1 on tumor growth in vivo. Briefly, 5×10^6^ K1/SOSTDC1 cells were injected subcutaneously into the left dorsal flank of each mouse, and the right side was inoculated with 5×10^6^ K1/Vector as a control. Tumor size was measured every 5 days, and the tumor volume was estimated. Thirty days after the injection, the mice were euthanized, and the tumors were removed and weighed. Then the excised tumors were fixed in 10% formalin for 24 h, embedded in paraffin wax and serially sectioned for 5 μm slides for immunohistochemistry staining. All experiments involving the use of animals were conducted in accordance with the recommendations in the Guide for the Care and Use of Laboratory Animals of the National Institutes of Health. The protocol was approved by the Institutional Animal Care and Use Committee of Sun Yat-sen University.

### Immunohistochemistry

Sections were deparaffinized, rehydrated, subjected to antigen retrieval and blocked. Then the tissues were incubated with the following antibodies: anti-SOSTDC1, anti-cyclin E2 (Abcam, Cambridge, MA), anti-cyclin A2 (Cell Signaling, Beverly, MA), and anti-Ki67 (Sigma-Aldrich, St. Louis, Missouri). The secondary antibodies were used. The immunohistochemical staining was evaluated in accordance with our previous report [34]. In brief, the proportion of tumor cells was graded as follows: 0 (no positive tumor cells), 1 (< 10% positive tumor cells), 2 (10-50% positive tumor cells), and 3 (> 50% positive tumor cells). The intensity of staining was determined as: 0 (no staining); 1 (weak staining = light yellow), 2 (moderate staining = yellow brown), and 3 (strong staining = brown). The staining index (SI) was calculated as staining intensity score × proportion of positive tumor cells, resulting in scores of 0, 1, 2, 3, 4, 6 and 9. An SI score of ≥ 4 was used to define tumors with high expression, and ≤ 3 as tumors with low expression.

### Bioinformatic analysis

We downloaded RNAseqV2 data of 51 pairs of thyroid tumors and their adjacent non-tumorous thyroid tissues, and 358 cases of thyroid cancers from The Cancer Genome Atlas (TCGA) website (https://tcga-data.nci.nih.gov/tcga) [35]. The fold differences in transcript abundance were compared by paired t test, using MultiExperiment Viewer (MeV) software (version 4.9, http://www.tm4.org/). Ingenuity Pathway Analysis (IPA) was conducted to identify the biological function mediated by these differentially expressed genes identified in low and high SOSTDC1 expression samples. Then IPA Upstream Regulator Analysis (URA) was performed to find out the altered upstream regulators that could be responsible for the observed expression changes. Gene Set Enrichment Analysis (GSEA) was carried out using GSEA 2.0.14 (http://www.broadinstitute.org/gsea/). The E2F target gene set was compiled from a review by Bracken *et al.* [36].

### Statistical analysis

All data are presented as means ± standard deviation (SD) of at least three independent experiments. Statistical analysis was performed using the SPSS17.0 software (SPSS Inc., Chicago, IL) and student t-test was used to compare the differences between two groups. A value of *P* < 0.05 was considered statistically significant.

## References

[1] Siegel R, Naishadham D, Jemal A (2013). Cancer statistics 2013 CA. Cancer J Clin.

[2] Russo D, Damante G, Puxeddu E, Durante C, Filetti S (2011). Epigenetics of thyroid cancer and novel therapeutic targets. J Mol Endocrinol.

[3] Nikiforov YE, Nikiforova MN (2011). Molecular genetics and diagnosis of thyroid cancer. Nat Rev Endocrinol.

[4] Lintern KB, Guidato S, Rowe A, Saldanha JW, Itasaki N (2009). Characterization of wise protein and its molecular mechanism to interact with both Wnt and BMP signals. J Biol Chem.

[5] Kassai Y, Munne P, Hotta Y, Penttila E, Kavanagh K, Ohbayashi N, Takada S, Thesleff I, Jernvall J, Itoh N (2005). Regulation of mammalian tooth cusp patterning by ectodin. Science.

[6] Beaudoin GM, Sisk JM, Coulombe PA, Thompson CC (2005). Hairless triggers reactivation of hair growth by promoting Wnt signaling. Proc Natl Acad Sci U S A.

[7] Collette NM, Yee CS, Murugesh D, Sebastian A, Taher L, Gale NW, Economides AN, Harland RM, Loots GG (2013). Sost and its paralog Sostdc1 coordinate digit number in a Gli3-dependent manner. Dev Biol.

[8] Shigetani Y, Howard S, Guidato S, Furushima K, Abe T, Itasaki N (2008). Wise promotes coalescence of cells of neural crest and placode origins in the trigeminal region during head development. Dev Biol.

[9] Clausen KA, Blish KR, Birse CE, Triplette MA, Kute TE, Russell GB, D'Agostino RB, Miller LD, Torti FM, Torti SV (2011). SOSTDC1 differentially modulates Smad and beta-catenin activation and is down-regulated in breast cancer. Breast Cancer Res Treat.

[10] Blish KR, Wang W, Willingham MC, Du W, Birse CE, Krishnan SR, Brown JC, Hawkins GA, Garvin AJ, D'Agostino RB, Torti FM, Torti SV (2008). A human bone morphogenetic protein antagonist is down-regulated in renal cancer. Mol Biol Cell.

[11] Ohshima J, Haruta M, Arai Y, Kasai F, Fujiwara Y, Ariga T, Okita H, Fukuzawa M, Hata J, Horie H, Kaneko Y (2009). Two candidate tumor suppressor genes, MEOX2 and SOSTDC1, identified in a 7p21 homozygous deletion region in a Wilms tumor. Genes Chromosomes Cancer.

[12] Rajkumar T, Vijayalakshmi N, Gopal G, Sabitha K, Shirley S, Raja UM, Ramakrishnan SA (2010). Identification and validation of genes involved in gastric tumorigenesis. Cancer Cell Int.

[13] Gopal G, Raja UM, Shirley S, Rajalekshmi KR, Rajkumar T (2013). SOSTDC1 down-regulation of expression involves CpG methylation and is a potential prognostic marker in gastric cancer. Cancer Genet.

[14] Manning AL, Dyson NJ (2012). RB: mitotic implications of a tumour suppressor. Nat Rev Cancer.

[15] Weinberg RA (1995). The retinoblastoma protein and cell cycle control. Cell.

[16] Gordon GM, Du W (2011). Conserved RB functions in development and tumor suppression. Protein Cell.

[17] Singh S, Johnson J, Chellappan S (2010). Small molecule regulators of Rb-E2F pathway as modulators of transcription. Biochim Biophys Acta.

[18] Blish KR, Clausen KA, Hawkins GA, Garvin AJ, Willingham MC, Turner JC, Torti FM, Torti SV (2010). Loss of heterozygosity and SOSTDC1 in adult and pediatric renal tumors. J Exp Clin Cancer Res.

[19] Barderas R, Mendes M, Torres S, Bartolome RA, Lopez-Lucendo M, Villar-Vazquez R, Pelaez-Garcia A, Fuente E, Bonilla F, Casal JI (2013). In-depth characterization of the secretome of colorectal cancer metastatic cells identifies key proteins in cell adhesion, migration, and invasion. Mol Cell Proteomics.

[20] Rawat A, Gopisetty G, Thangarajan R (2014). E4BP4 is a repressor of epigenetically regulated SOSTDC1 expression in breast cancer cells. Cell Oncol (Dordr).

[21] Gong D, Pomerening JR, Myers JW, Gustavsson C, Jones JT, Hahn AT, Meyer T, Ferrell JE (2007). Cyclin A2 regulates nuclear-envelope breakdown and the nuclear accumulation of cyclin B1. Curr Biol.

[22] Zhong WB, Hsu SP, Ho PY, Liang YC, Chang TC, Lee WS (2011). Lovastatin inhibits proliferation of anaplastic thyroid cancer cells through up-regulation of p27 by interfering with the Rho/ROCK-mediated pathway. Biochem Pharmacol.

[23] Yasmeen A, Berdel WE, Serve H, Muller-Tidow C (2003). E- and A-type cyclins as markers for cancer diagnosis and prognosis. Expert Rev Mol Diagn.

[24] Gudas JM, Payton M, Thukral S, Chen E, Bass M, Robinson MO, Coats S (1999). Cyclin E2, a novel G1 cyclin that binds Cdk2 and is aberrantly expressed in human cancers. Mol Cell Biol.

[25] Payton M, Scully S, Chung G, Coats S (2002). Deregulation of cyclin E2 expression and associated kinase activity in primary breast tumors. Oncogene.

[26] Caldon CE, Sergio CM, Schutte J, Boersma MN, Sutherland RL, Carroll JS, Musgrove EA (2009). Estrogen regulation of cyclin E2 requires cyclin D1 but not c-Myc. Mol Cell Biol.

[27] Desmedt C, Ouriaghli FE, Durbecq V, Soree A, Colozza MA, Azambuja E, Paesmans M, Larsimont D, Buyse M, Harris A, Piccart M, Martiat P, Sotiriou C (2006). Impact of cyclins E, neutrophil elastase and proteinase 3 expression levels on clinical outcome in primary breast cancer patients. Int J Cancer.

[28] Sieuwerts AM, Look MP, Meijer-van Gelder ME, Timmermans M, Trapman AM, Garcia RR, Arnold M, Goedheer AJ, de Weerd V, Portengen H, Klijn JG, Foekens JA (2006). Which cyclin E prevails as prognostic marker for breast cancer? Results from a retrospective study involving 635 lymph node-negative breast cancer patients. Clin Cancer Res.

[29] Zhao Z, Liu J, Wang C, Wang Y, Jiang Y, Guo M (2014). MicroRNA-25 regulates small cell lung cancer cell development and cell cycle through cyclin E2. Int J Clin Exp Pathol.

[30] Gordon GM, Du W (2011). Targeting Rb inactivation in cancers by synthetic lethality. Am J Cancer Res.

[31] Yamauchi K, Yang M, Hayashi K, Jiang P, Yamamoto N, Tsuchiya H, Tomita K, Moossa AR, Bouvet M, Hoffman RM (2008). Induction of cancer metastasis by cyclophosphamide pretreatment of host mice: an opposite effect of chemotherapy. Cancer Res.

[32] Guan H, Cai J, Zhang N, Wu J, Yuan J, Li J, Li M (2012). Sp1 is upregulated in human glioma, promotes MMP-2-mediated cell invasion and predicts poor clinical outcome. Int J Cancer.

[33] Guan H, Wei G, Wu J, Fang D, Liao Z, Xiao H, Li M, Li Y (2013). Down-regulation of miR-218-2 and its host gene SLIT3 cooperate to promote invasion and progression of thyroid cancer. J Clin Endocrinol Metab.

[34] Guan H, Liang W, Liu J, Wei G, Li H, Xiu L, Xiao H, Li Y (2014). Transmembrane Protease Serine 4 Promotes Thyroid Cancer Proliferation via CREB Phosphorylation. Thyroid.

[35] Cancer Genome Atlas Research N (2008). Comprehensive genomic characterization defines human glioblastoma genes and core pathways. Nature.

[36] Bracken AP, Ciro M, Cocito A, Helin K (2004). E2F target genes: unraveling the biology. Trends Biochem Sci.

